# Direct repeat-mediated DNA deletion of the mating type *MAT1-2* genes results in unidirectional mating type switching in *Sclerotinia trifoliorum*

**DOI:** 10.1038/srep27083

**Published:** 2016-06-03

**Authors:** Liangsheng Xu, Teresa M. Jardini, Weidong Chen

**Affiliations:** 1 Department of Plant Pathology, Washington State University, Pullman, WA 99164, USA; 2 USDA-ARS, Grain Legume Genetics and Physiology Research Unit, Washington State University, Pullman, WA 99164, USA

## Abstract

The necrotrophic fungal pathogen *Sclerotinia trifoliorum* exhibits ascospore dimorphism and unidirectional mating type switching - self-fertile strains derived from large ascospores produce both self-fertile (large-spores) and self-sterile (small-spores) offsprings in a 4:4 ratio. The present study, comparing DNA sequences at *MAT* locus of both self-fertile and self-sterile strains, found four mating type genes (*MAT1-1-1*, *MAT1-1-5*, *MAT1-2-1* and *MAT1-2-4*) in the self-fertile strain. However, a 2891-bp region including the entire *MAT1-2-1* and *MAT1-2-4* genes had been completely deleted from the *MAT* locus in the self-sterile strain. Meanwhile, two copies of a 146-bp direct repeat motif flanking the deleted region were found in the self-fertile strain, but only one copy of this 146-bp motif (a part of the *MAT1-1-1* gene) was present in the self-sterile strain. The two direct repeats were believed to be responsible for the deletion through homologous intra-molecular recombination in meiosis. Tetrad analyses showed that all small ascospore-derived strains lacked the missing DNA between the two direct repeats that was found in all large ascospore-derived strains. In addition, heterokaryons at the *MAT* locus were observed in field isolates as well as in laboratory derived isolates.

Fungi have evolved a remarkable diversity of reproductive strategies adapting to the changing environments. Most fungi are able to reproduce both sexually and asexually. Under favorable environmental conditions, fungi may preferentially clonally expand by asexual reproduction. However, when the environmental conditions become adverse some fungi will reproduce sexually to generate genetic variation via recombination and/or resilient surviving structure in the population to enhance and ensure their survival.

In most filamentous ascomycetes, sexual reproduction is mainly controlled by two idiomorphs, *MAT1-1* and *MAT1-2*^1^,^2^. *MAT1-1* encodes a transcription factor with an alpha-1 domain. *MAT1-2* encodes a protein with a high mobility group (HMG) DNA-binding domain. The mating-type genes are dissimilar in sequence but found at the same locus on the chromosome and generally flanked by the DNAlyase (*APN2*) and Cytoskeleton assembly (*SLA2*). Depending on species, some fungi may contain additional genes in the *MAT* locus.

Several modes of sexual reproductions in ascomycetes have been reported including homothallic (self-fertile), heterothallic (requires a mating partner, one locus with two alleles), pseudohomothallic (a single ascospore with two nuclei of opposite mating type allele)^2^,^3^,^4^,^5^, dual mating (isolates can mate with both *MAT1-1* and *MAT1-2* tester strains), and unidirectional mating type switching (self-fertile strains produce both self-fertile and self-sterile strains)^2^,^3^,^6^,^7^.

The ascomycetous genus *Sclerotinia* has three economically important species including *Sclerotinia minor*, *S. sclerotiorum* and *S. trifoliorum*. *S. minor* and *S. sclerotiorum* are homothallic, in which single ascospore-derived strains are able to self-fertilize under controlled laboratory conditions, and produce ascospores that are fairly uniform in size. However, the ascospores of *S. trifoliorum* always show size dimorphism with large and small ascospores (4:4 segregation)^8^,^9^. Ascospore dimorphism was reported in *S. trifoliorum* in a taxonomical context as early as in 1954, but was initially considered to be due to heterokaryosis^10^. It was in 1979 that ascospore size dimorphism was considered to be a character of the species^11^. Uhm and Fujii^8^ showed that strains derived from large ascospores are self-fertile (designated L mating type). L mating type strains can produce large and small ascospores with 4:4 segregation^8^. In contrast, strains derived from small ascospores are self-sterile (designated S mating type). S mating type strains, although self-sterile, can be fertile when spermatized with microconidia from L mating type strains, and produce large and small ascospores in 4:4 segregation^8^,^9^. This phenomenon of a self-fertile strain giving rise to both self-fertile and self-sterile strains in *S. trifoliorum*, as well as in other fungal genera, was termed unidirectional mating type switching^12^. Beside in *S. trifoliorum*, unidirectional mating type switching occurs in three other fungal genera all in the class Sordariomycetes: *Ceratocystis, Chromocrea and Glomerella*^8^,^12^,^13^,^14^,^15^. Compared to the other sexual reproduction systems in fungi, such as homothallism and heterothallism, the mechanisms of unidirectional mating type switching are less studied. Recent studies have shed some light on the possible mechanisms. Witthuhn *et al.*^15^, using PCR specific for the HMG-DNA binding domain, showed that loss of *MAT1-2* idomorph was associated with mating type switching in *Ceratocystis* spp. Wilken *et al.*^14^ completely characterized the MAT locus sequences and demonstrated that two 260-bp direct repeats flanking a 3581-bp DNA region caused deletion of this flanked DNA region including *MAT1-2* genes in *C. fimbriata*. They provided the first sequence evidence that direct repeat-mediated deletion results in loss of *MAT1-2* genes and in self sterility^14^.

The structure of the mating type locus in *S. minor* and *S. sclerotiorum* has been characterized^16^,^17^. Although both *S. sclerotiorum* and *S. minor* are homothallic, they have two mating type alleles differentiated by a natural inversion of about 3.6-kb DNA, designated as Inv− and Inv+ *MAT* alleles^16^,^17^. Each allele contains four *MAT* genes including *MAT1-1-1*, *MAT1-1*-5, *MAT1-2-1* and *MAT1-2-4*. Compared to the Inv− *MAT* allele, about a 3.6-kb region was inverted in the Inv+ *MAT* allele, which affected orientation of three of the four *MAT* genes and caused truncation at the 3′ end of *MAT1-1-1* and inversion of *MAT1-2-4* and *MAT1-2-1*. However, the *MAT* locus structure in *S. trifoliorum* is not known, and it was speculated that the mating type switch in *S. trifoliorum* was related the *MAT* gene inversion found in *S. sclerotiorum* and *S. minor*^16^,^17^.

*Sclerotinia trifoliorum* is not only unique among *Sclerotinia* spp in producing dimorphic ascospores, but also an important plant pathogens of many cool season legume crops such as alfalfa, clover and chickpea^8^,^11^. In order to determine whether the mating type switching in *S. trifoliorum* is caused by mating type allele inverstion or by DNA deletion, this study was carried out with three objectives: 1) characterize the structure of the *MAT* locus in the L and S mating types of *S. trifoliorum* and uncover the possible mechanisms of the unidirectional mating type switching; 2) compare *MAT* locus with that of *S. minor* and *S. sclerotiorum* to better understand the evolution of *MAT* alleles in *Sclerotinia*; and 3) develop a screening technique for mating type determination of *S. trifoliorum*. The results provide further insight into the molecular basis of unidirectional mating type switching and the DNA affected during this evolutionary process.

## Results

### The structure of the *MAT* locus in *S. trifoliorum* self-fertile L mating type most resembles that of *MAT* inversion positive strains of *S. minor* and *S. sclerotiorum*

**Figure 1:**
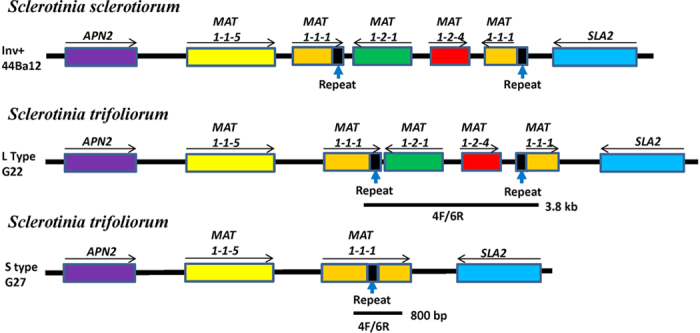
Schematic drawing showing the arrangements and orientations of genes in the mating type locus of *S. trifoliorum* self-fertile L mating type (G22) and self-sterile S mating type (G27) isolates. The *Sclerotinia sclerotiorum* Inv+ *MAT* allele (44Ba12, adapted from Chitrampalam *et al.*^17^) is included for comparison. Note the different orientations of the 3′ fragment of *MAT1-1-1* gene. The DNA region amplified using the diagnostic primer pair 4F and 6R is indicated which will amplify 3800 bp and 800 bp in the L and S mating types, respectively. Diagram not drawn to scale.

The genome of *S. trifoliorum* isolate 06CWM-G22 (hereafter G22) was sequenced to 53× coverage using the PacBio platform, and the assembled genome of about 39.8 Mb consisted of 20 contigs with N50 length at 2.23 Mb. Telomeres could be detected at both ends of many of the contigs, suggesting the contigs likely represent chromosomes. Contig 3 of about 3.14 Mb was identified to contain the *MAT* locus (about 16 kb) based on CLC Genomics Workbench searches using the *MAT* allele (JQ815884) of *S. sclerotiorum* as query. Five open reading frames (ORF) were predicted within this locus using AUGUSTUS. The first ORF encodes a protein with a high level of similarity to the *APN2*. The products predicted for the other four complete ORFs showed similarity to *MAT1-1*-5, *MAT1-2-1*, *MAT1-2-4* and *SLA2*, and an ORF with high similarity to *MAT1-1-1* was divided into two parts by *MAT1-2-1* and *MAT1-2-4* (Fig. 1). The gene order and orientation in the *MAT* locus of self-fertile isolate mostly resemble those reported for the Inv+ strains of *S. minor* and *S. sclerotiorum*, including truncation of the *MAT1-1-1* gene. The only difference is in the translational orientation of the truncated 3′ fragment of the *MAT1-1-1*; it is in the same orientation as the 5′ fragment of *MAT1-1-1* in *S. trifoliorum*, but in an opposite orientation in *S. minor* and *S. sclerotiorum* (Fig. 1)^16^,^17^.

### A 2966-bp DNA region was missing in the *MAT* locus of the self-sterile S mating type

The overlapping 13 pairs of PCR primers designed based on genome sequence were used to amplify DNA fragments from genomic DNA of strains G22 (self-fertile) and 06CWM-G27 (self-sterile, hereafter G27) covering the entire *MAT* locus including the flanking genes *APN2* and *SLA2* (see Supplementary Fig. S1). Each pair of the PCR primers was able to amplify expected PCR product from strain G22. Sequences of the PCR products were confirmed by Sanger sequencing. The assembled sequence from the 13 overlapping PCR products from G22 was 16.3 kb long and showed 100% sequence identity to that region in the draft genome, suggesting the quality of the draft genome is highly reliable. However, only 10 of the 13 primer pairs produced expected fragments in the PCR amplifications from strain G27 and the nucleotide sequences of the amplified PCR products were identical to those of strain G22. The remaining three primer pairs (4F/4R, 5F/5R and 6F/6R) did not produce any PCR products from the self-sterile isolate G27. When PCR primers 4F and 6R were paired in PCR, a single PCR product of about 800 bp was amplified, which was subsequently sequenced. This primer pair would produce a PCR product of about 3800 bps in strain G22. The assembled sequence for the G27 *MAT* locus was 13.9 kb long, 2966-bp shorter than that found in the strain G22, and contained four predicted ORFs, which showed high similarity to APN2, *MAT1-1*-5, *MAT1-1-1* and SLA2 (Fig. 1). The entire *MAT1-2-1* and *MAT1-2-4* genes were missing along with the deleted 2966-bp DNA region (Fig. 1). The DNA sequences of the *MAT* locus of self-fertile and self-sterile strains were deposited at GenBank and assigned accession numbers KU726096 and KU726097, respectively.

### A 146-bp repeat motif flanked the missing DNA region in the self-fertile L mating type strain

**Figure 2:**
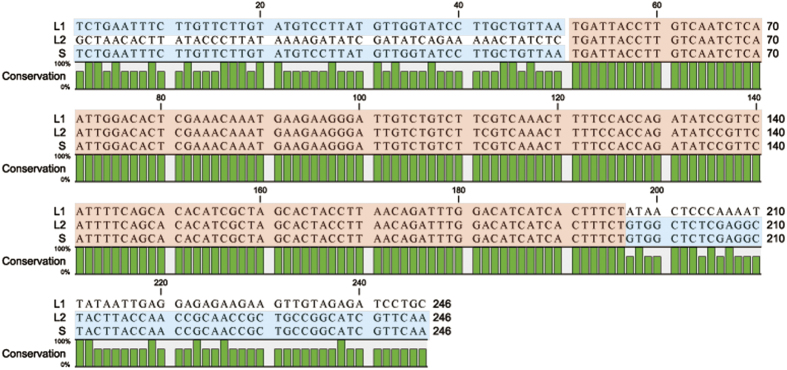
Alignment of the three repeat sequences plus 50 bp upstream and 50 bp downstream at the *MAT* locus *Sclerotinia trifoliorum*, the first copy (L1) and second copy (L2) of the repeats in the L *MAT* allele (self-fertile) and the single copy (S) of the repeat in the S *MAT* allele (self-sterile). The repeat sequences L1, L2 and S (in red blocks) are 100% identical. The 50 bp upstream of the S repeat is 100% identical to that upstream of the L1 repeat, and the 50 bp downstream of the S repeat is 100% identical to that downstrwam of the L2 repeat (identical sequences outside of the repeat sequences are in blue blocks).

Comparison between the two *MAT* alleles in the *S. trifoliorum* strains G22 and G27 revealed a 2966-bp region was missing in the *S. trifoliorum* strain G27, and the missing DNA region contained the entire *MAT1-2-1* and *MAT1-2-4* genes. RepFind analysis with a p-value of 0.00 identified a 146-bp direct repeat in the *S. trifoliorum* G22 *MAT* locus that appeared twice and located at the boundaries of the deletion region. The first copy of the 146-bp repeat is located in and a part of the *MAT1-1-1* gene, whereas the second copy of the repeat is located in a non-coding region. This repeat sequence appeared only once in the S allele strain G27 and is in the *MAT1-1-1* gene. The sequence immediately upstream of the repeat motif in the S allele was 100% identical to that upstream of the first copy of the repeat motif in the L allele, whereas the sequence immediately downstream of the repeat motif in the S allele was 100% identical to that downstream of the second copy of the repeat motif in the L allele (Fig. 2). These sequence comparisons strongly suggest that the deletion in the S allele was resulted from intra-molecular homogolous recombination anchored at the two repeats in the L allele, similar to that reported for *C. fimbriata*^14^.

**Figure 3:**
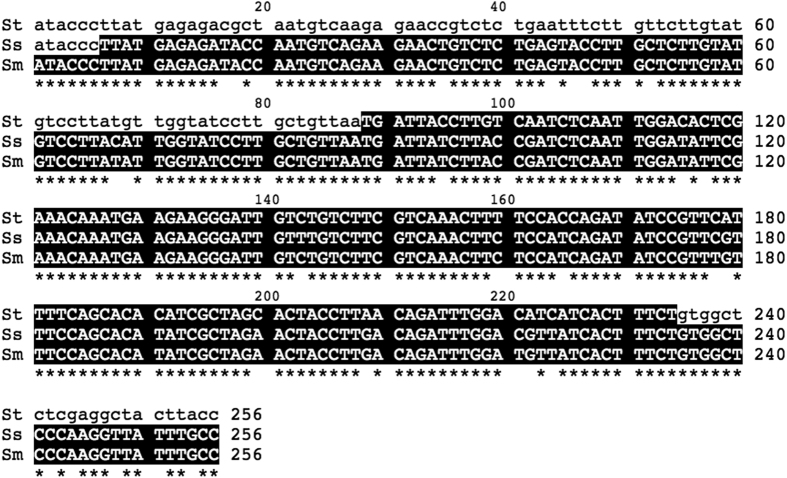
Alignment of the direct repeat motif region of self-sterile isolate of *Sclerotinia trifoliorum* (St, strain G27, 146 bases) with the first copy of the inverted repeat of *S. sclerotiorum* (Ss, strain SS44Ba1, 250 bases) and *S. minor* (Sm, strain SM1, 256 bases). Repeat motifs are in black background and *indicates identical nucleotide among the three species.

Previous studies showed that 250-bp and 256-bp inverted repeat sequences were found in the *S. sclerotiorum* and *S. minor MAT* locus, respectively^16^,^17^. Alignment of these repeat sequences from the three Sclerotinia species showed the repeat motif in *S. trifoliorum* was highly similar to part of the other two repeat sequences, although they were different in length (Fig. 3). The repeat motif in *S. trifoliorum* showed 88.4 and 89.0% identical in nucleotides with the respective repeat motifs in *S. minor* and *S. sclerotiorum*, respectively.

### Characterization of *MAT* genes in *S. trifoliorum*

The *MAT1-1*-5 gene was 1306-bp long and was identical in both strains G22 and G27 (Table 1). The *MAT1-1-1* was 1212-bp long and continuous in the self-sterile strain G27, but was split into two pieces (5′ fragment, 677-bp, and 3′ fragment, 601-bp) in the self-fertile strain G22. The *MAT1-2-1* and *MAT1-2-4* ORFs, present only in the self-fertile strain G22, were 1098 and 879 bp long, respectively. All the *MAT* genes of *S. trifoliorum* differed from their respective *MAT* genes in *S. minor* strain SM1^16^, *S. sclerotiorum* strains 1980^18^ and 44Ba1^17^ and *B. cinerea* strain T4^18^. Comparison between the *MAT* genes of *S. trifoliorum* to their corresponding *MAT* genes in *S. minor, S. sclerotiorum and B. cinerea* indicated 43% to 95% nucleotide identity depending on the *MAT* gene (Table 1). *MAT1-1-1* was 90, 89 and 77% identical to the corresponding genes in *S. minor, S. sclerotiorum* and *B. cinerea*, respectively. *MAT1-1*-5 was 95, 95 and 77% identical to the corresponding genes in *S. minor, S. sclerotiorum and B. cinerea*, respectively. *MAT1-2-1* was 94, 94 and 78% identical to the genes in *S. minor, S. sclerotiorum and B. cinerea*, respectively. Among the four *MAT* genes, *MAT1-2-4* was the most divergent with the lowest sequence identity (43%) to *B. cinerea* homologs. *MAT1-1*-5, *MAT1-2-1* and *MAT1-2-4* in isolate G22 contains 3, 2 and 1 introns, respectively, and *MAT1-1*-5 and *MAT1-1-1* in isolate G27 contains 3 and 2 introns, respectively (Table 1). Sequencing RT-PCR products showed that the intron sequences were absent in the RT-PCR products.

### Analysis of the intergenic spacer regions between *MAT* genes in *Sclerotinia spp*

The arrangement of *MAT* genes and their translation directions in the L mating type strain of *S. trifoliorum* were most similar to those of the Inv+ *MAT* allele of *S. minor* and *S. trifoliorum*. Therefore, the intergenic regions of *S. trifoliorum MAT* locus were compared with those in the Inv+ *MAT* allele of *S. minor* and *S. trifoliorum.* Except for the spacer region between *MAT1-2-1* and *MAT1-2-4*, significant difference in length in all other *MAT* intergenic regions were found among the three *Sclerotinia spp.* (Table 2). Similar to *S. sclerotiorum* and *S. minor*, the intergenic region between *APN2* and *MAT1-1*-5 (2917 bp) is the longest among the noncoding intergenic regions at the *MAT* locus in *S. trifoliorum*, and it is 20 and 1318 bp longer than its corresponding regions in *S. sclerotiorum* and *S. minor*, respectively. The intergenic region between *MAT1-1*-5 and the 5′ fragment of *MAT1-1-1* is 2851 bp, which is 2445 bp longer than its homologue in *S. sclerotiorum* and *S. minor*. A 12-bp intergenic region between the 5′ fragment of *MAT1-1-1* and *MAT1-2-1* in strain G22 were identified, but no such a spacer region was found in the Inv+ *MAT* allele of *S. sclerotiorum* and *S. minor*. The intergenic region between *MAT1-2-4* and 3′ fragment of *MAT1-1-1* is 177-bp long, and 330-bp shorter than its homologue in *S. sclerotiorum* and *S. minor*. The intergenic region between the 3′ fragment of *MAT1-1-1* and *SLA2* is 1292-bp long, and 676 and 668 bp longer than its homologue in *S. sclerotiorum* and *S. minor*, respectively.

### Comparison of *MAT* proteins

The analysis of amino acid sequences indicated that *MAT* proteins in *S. trifoliorum* were 73–92% identical to the respective proteins in *S. min*or, 64–91% identical to those of the respective proteins in *S. sclerotiorum* and 51-77% identical to the corresponding proteins in *B. cinerea*. Among the four *MAT* proteins of *S. trifoliorum*, *MAT1-2-4* protein exhibited the most divergent with lowest identity (51–74%) to the homologs in *S. min*or, *S. sclerotiorum*, and *B. cinerea*.

Comparison of the conserved alpha-box (for *MAT1-1-1*) and HMG-box (for *MAT1-2-1*) of *S. trifoliorum* to the respective domains in *S. minor*, *S. sclerotiorum*, and *B. cinerea* revealed that the HMG-box in *S. trifoliorum* was more similar than the alpha-box to the corresponding domains in other species (Supplementary Fig. S2).

### Transcription analysis of *MAT* genes in *S. trifolorium*

**Figure 4:**
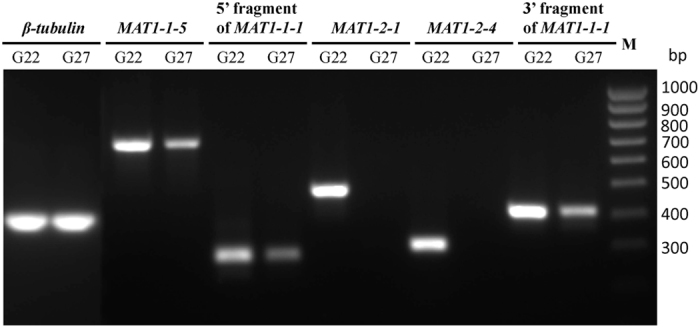
Expression of *MAT* genes of self-fertile isolate G22 and self-sterile isolate G27 of *Sclerotinia trifoliorum.* Primer sequences and expected product sizes are shown in Supplementary Table S3 and sequencing of the RT-PCR products showed no intron sequences, suggesting the products were from RNA only. Lane M is DNA ladder 100-bp Hyperladder.

RT-PCR was carried out to detect expression of the *MAT* genes in *S. trifoliorum* strains G22 and G27. Three of the five primer pairs used in the RT-PCR each spanned an intron (Supplementary Table S3), facilitated detection of potential DNA contamination in the RNA preparations. Sequencing of the RT-PCR products did not detect the intron sequences suggesting that there was no DNA contamination. The results showed all four *MAT* genes (*MAT1-1-1*, *MAT1-1*-5, *MAT1-2-1* and *MAT1-2-4*) in *S. trifoliorum* strain G22 were expressed (Fig. 4). Even the two split fragments of *MAT1-1-1* gene were expressed although the 3′ fragment of *MAT1-1-1* lacked an in-frame start codon. Expression of *MAT1-1*-5 and *MAT1-1-1* were also detected in the self-sterile strain G27 of *S. trifoliorum*. However, no transcripts of *MAT1-2-1* and *MAT1-2-4* genes were detected in the self sterile strain G27 (Fig. 4).

### *MAT* allele determination by PCR and association of the *MAT* alleles with ascospore size

**Figure 5:**
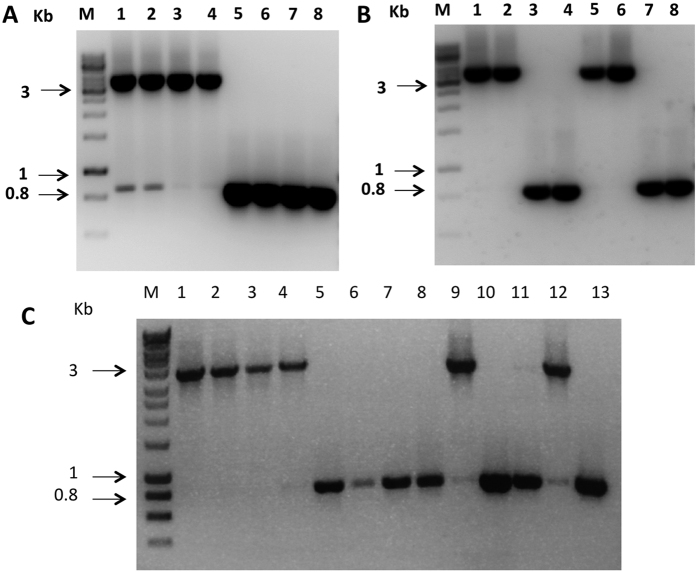
PCR detection of the *MAT* alleles of *Sclerotinia trifoliorum* isolates using the specific primers 4F and 6R. (**A**) Mating type alleles of single-ascospore strains from an ascus of isolate 06CWM-G22 with 4 large and 4 small ascospores distriution. Lanes 1, 2, 3 and 4 are strains derived from large ascospores, and lanes 5, 6, 7 and 8 are strains derived from small ascospores. (**B**) Mating type alleles of single ascospore isolates from the an ascus of isolate 06CWM-G34. Lanes 1, 2, 5 and 6 are strains derived from large ascospores, and lanes 3, 4, 7 and 8 are strains derived from small sacospores. (**C**) Mating type alleles of *S. trifioliorum* field isolates. Lanes in 1-13 are 06CWM-G22, 06CWM-G48, 06CWM-G2, 06CWM-G55, 06CWM-F9, 06CWM-F6, 06CWM-F2, 05WM21, 06CWM-G7, 06CWM-G23, 06CWM-D5, 06CWM-G39 and 06CWM-G27, respectively.

The two primers (4F and 6R) that flank the deletion region were used to detect and differentiate the L and S *MAT* alleles in single ascospore isolates and field isolates. Ascospores in two asci (each from a different field strain) were determined to be either large or small spores microscopically at time of ascus dissection. The large and small ascospores were in 4:4 arrangement in one ascus from strain G22 and in 2:2:2:2 arrangement in the other ascus from strain G34. Without exception, PCR reactions with primers 4F and 6R always produced the small amplicon of 800 bps (S *MAT* allele) in the strains derived from the small ascospores, and the large amplicon of 3800 bp (L *MAT* allele) in the strains derived from large ascospores (Fig. 5AB). Occasionally isolates with L *MAT* allele also showed a faint PCR product of the S allele, showing potential heterokaryon at the *MAT* locus. The nature of the faint band as to its origin remains to be investigated.

Additionally, the 4F and 6R PCR primers were applied to 22 field isolates to test their applicability to differentiate the *MAT* alleles in isolates of different genetic backgrounds. Nine of the 22 isolates produced the 0.8 kb product and are S mating type. Thirteen isolates produced the 3.8 kb band, suggesting they are L mating type isolates (Fig. 5C; see Supplementary Table S1). And five of these 13 solates also showed a faint 0.8 kb band, which mean these isolates are likely heterokaryotic with respect to the *MAT* locus (Fig. 5, see Supplementary Table S1). The heterokaryons are self-fertile and have the phenotype of the L mating type. The field isolates could be unambiguously differentiated into either the L or the S mating types using PCR with primers 4F and 6R.

## Discussion

The *MAT* loci in two homothallic *Sclerotinia* species *S. minor* and *S. sclerotiorum*, close relatives of *S. trifoliorum*, were recently characterized^16^,^17^. Two mating type (*MAT*) alleles, inversion negative (Inv−) and inversion positive (Inv+) have been reported in these two species. They have typical features of *MAT* locus in homothallic Pezizomycotinia, in which two idomorphs, *MAT1-1* with the alpha domain and *MAT1-2* with HMG-domain, are present in tandem in a single *MAT* locus that is flanked by *APN2* and *SLA2*. In addition, the *MAT* loci in these two species carry two newly discovered *MAT* genes, *MAT1-1-5* and *MAT1-2-4*, which are homologues of *MAT1-1* and *MAT1-2*, respectively^18^. The present study showed the organization of *MAT* allele in *S. trifoliorum* G22 was identical to that in the Inv+ *MAT* allele of both *S. sclerotiorum* and *S. minor*, except for the orientation of the truncated 3′ fragment of *MAT1-1-1*. In *S. trifoliorum* strain G22, the orientation of the 3′ fragment of *MAT1-1-1* is in the same direction as the 5′ fragment of *MAT1-1-1*. In contrast, the 3′ fragment and the 5′ fragment of the *MAT1-1-1* gene were in opposite orientation in the Inv+ *MAT* allele of both *S. sclerotiorum* and *S. minor*^16^,^17^. Depending on the *MAT* genes, 89–95% nucleotide identity was observed among the three *Sclerotinia* spp. Another similarity in the *MAT* locus among the *Sclerotinia* spp. was the presence of repeat motifs of the same origin with significant sequence identities (Fig. 3) and the repeat motif is part of the *MAT1-1-1* gene sequence in all three *Sclerotinia* spp.

Despite these similarities, there are significant differences in repeat motif orientation, in gene sequences and in the noncoding spacer regions at the *MAT* locus among the *Sclerotinia* spp, although the functions of noncoding spacer regions during the evolution of mating locus are unknown. The major difference in the *MAT* locus among the *Sclerotinia* spp. is the orientation of the repeat motifs. In *S. minor* and *S. sclerotiorum*, the repeat motifs are inverted (in opposite direction), whereas in *S. trifoliorum*, the repeat motifs are direct repeat (in the same direction). During meiosis via recombination, inverted repeats would cause inversion of the DNA region between the repeats^16^,^17^, whereas direct repeats would cause deletion of the DNA region between the repeats^14^. The situation in *S. trifoliorum* is very similar to that recently reported for the mating type switching mechanism in *Ceratocystis fimbriata* by Wilken *et al.*^14^. Wilken *et al.* showed that two 260-bp direct repeats flanking the deleted DNA region in self-fertile isolates of *C. fimbriata*, and the DNA region between the two repeats along with one copy of the repeat motif was missing in the self-sterile isolates. Thus the unidirectional mating type switching in *S. trifoliorum* is caused by the same molecular mechanisms as reported in *C. fimbriata*: direct repeat-mediated DNA deletion causing loss of MAT-1-2 genes results in self-sterility.

Despite of the same molecular mechanism for the unidirectional mating type switching in both *C. fimbriata* and *S. trifoliorum*, this unidirectional mating type switching phenotype has obviously evolved independently. First, the repeat sequences are completely different and there are no sequence similarities between the repeat motifs of *C. fimbriata* and *S. trifoliorum*. Second, the locations of the repeat sequences are totally different. The repeat motif is located in and a part of the *MAT1-1-1* gene in *S. trifoliorum*, whereas they are located in non-coding regions in *C. fimbriata*, Finally, the genes, gene orientations and flanking genes are different. The *MAT* locus of *S. trifoliorum* possesses *MAT1-1*-5 and *MAT1-2-4*, unique for *Sclerotinia* and *Botrytis*, whereas the *MAT* locus of *C. fimbriata* possesses *MAT1-1*-2. The *MAT* locus in *S. trifoliorum* is flanked by *APN2* and *SLA2* genes, typically found in many Ascomycetes, whereas the *MAT* locus in *C. fimbriata* is downstream of *APN2* and *SLA2* genes^14^.

**Figure 6:**
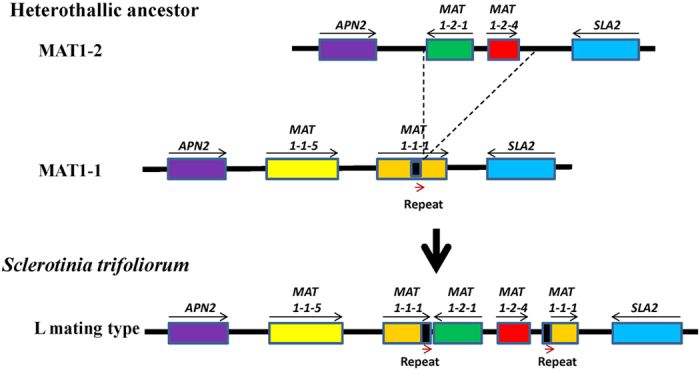
Proposed evolution of the self-fertile L *MAT* allele (homothallic) of the *MAT* locus in *S. trifoliorum* from a hypothetical hetherothallic ancestor through a one-step mutation (a crossover).

Homothallism and heterothallism are the two most common strategies of sexual reproduction in fungi. Different hypotheses exist as to which came first^1^,^2^. In Sclerotinacae, an early hypothesis suggested that heterothallic *Botrytis cinerea* evolved from homothallic *S. sclerotiorum* through four step mutations^18^. Chitrampalam *et al.*^17^ proposed a more parsimonious three-step mutation for evolution of homothallic *Sclerotinia* spp. from heterothallic *Botrytis*. Understanding the *MAT* locus structures in *S. trifoliorum* helped shed more light on this. The structure of L allele of *S. trifoliorum MAT* locus most resembles the structure of the Inv+ *MAT* allele in *S. minor* and *S. sclerotiorum*. However, the structure of S allele of *S. trifoliorum* is the same as in the MAT1-1 allele (idiomorph) in *Botrytis cinerea*. Imagining an ancestral heterothallic species with *MAT* locus similar to *B. cinerea*, a one-step mutation, a crossover would result in insertion of the *MAT1-2* allele (idiomorph) into *MAT1-1* idiomorph and truncate *MAT1-1-1* gene, forming a structure as the L allele of *S. trifoliorum* (Fig. 6) and the Inv+ *MAT* allele of *S. minor* and *S. sclerotiorum*. Our data support the hypothesis that homothallic *Sclerotinia* spp evolved from an ancestral heterothallic species.

Just like in *S. sclerotiorum* and *S. minor*, the *MAT* genes were constitutively expressed during vegetative growth. Thus the *MAT* genes may have other functions besides sexual reproduction. In *S. trifoliorum*, there is a clear pleiotrophic effect on ascospore size. Lack of the MAT1*-2* genes resulted in ascospores of a smaller size. Thus the ascospore size is correlated to the genotype in the spore itself, not the genotype of the parent strain. We compared 20 isolates each derived from large ascospores and small ascospores for mycelial growth and sclerotial production on PDA, but did not observe difference between the two types (unpublished data). However, a study in *Ceratocystis albifundus* showed that self-sterile isolates (without *MAT1-2* idiomorph) have lower fitness in germination, growth rate and pathogenicity than self-fertile isolates^19^. An earlier study suggested that the two spore types of *S. trifoliorum* may differ in initial germination rate^8^. Whether the *MAT* genes have any other pleiotrophic effect in *S. trifoliorum* requires further investigation. Although the *MAT1-1-1* gene in the L allele was truncated and divided by the *MAT1-2* genes, and its 3′ fragment lacked an in-frame start codon, both the two divided fragments were successfully transcribed like the other three intact *MAT* genes (Fig. 4). Similar results were reported in *S. minor* and *S. sclerotiorum*, in which the 3′ fragment of the truncated *MAT1-1-1* gene was also expressed. Whether the *MAT1-1-1* gene could still be functional without expressing its 3′ fragment downstream of the alpha box in a homothallic strain is still unknown.

In this study we also observed heterokayons at the *MAT* locus (presence of both L and S alleles) in field isolates as well as in laboratory-derived single ascospore isolates (Fig. 5). Interestingly, in all cases, it was the L allele showing the stronger band than the S allele. This unequal PCR product intensity was unlikely due to preferential amplification because the weaker band was always the S allele that is smaller in size than the L allele, and smaller sized PCR product would be preferentially amplified. Heterokaryon at *MAT* locus has also been reported in many filamentous fungi including *S. minor* and S. *sclerotiorum*, *Botryotinia fuckeliana*, and *S. homoeocarpa*^16^,^17^,^20^,^21^,^22^,^23^,^24^. Especially, the heterokaryosis during the vegetative phase was commonly found in the most isolates of *Cryphonectria parasitica* collected from the field^22^,^23^. Formation of heterokaryons in *S. sclerotiorum* under nonrestrictive conditions was demonstrated before^25^, and more than 50% of field isolates of *S. minor* and *S. sclerotiorum* were found to be heterokaryotic at the *MAT* locus^16^,^24^. However, heterokaryons were not observed in microsatellite loci among many isolates of *S. sclerotiorum*^26^,^27^. Future study should be directed at determining whether such repeat sequence-mediated DNA deletion or DNA inversion through intramolecular recombination observed during meosis could also occur during mitosis at low frequency, which could have significant implications in interpreting population genetic data.

## Material and Methods

### Strains and nucleic acid extraction

*S. trifoliorum* isolates were collected from stems of infected chickpea plants from various locations in central California, USA (see Supplementary Table S1)^28^. The isolates were maintained at 20 °C on PDA. Long-term storage stock was maintained at −20 °C as sclerotia. Mycelia of 3 to 4 day old culture were harvested from colonies grown on cellophane-covered PDA plate and used for nucleic acid extraction immediately or stored at −20 °C until use. Genomic DNA was extracted using the Qiagen Plant DNA extraction kit (Qiagen) and quantified using a ND-1000 Nanodrop spectrometer (NanoDorp Technologies). The working concentration of DNA for PCR amplification was adjusted to10 ng/μL with sterilized distilled water. Total RNA was extracted from the mycelium of isolates G22 and G27 using the Qiagen RNeasy^TM^ Plant Mini Kit following the manufacturer’s protocol and stored at −20 °C until use.

### Tetrad analyses

Apothecia were produced from sclerotia of L mating type strains 06CWM-G22 and 06CWM-G34 following the protocol described by Njambere *et al.*^28^. Asci in an apothecium were collected by crashing the apothecia and diluting in 1 mL water, then spread on one end of a microscope slide coated with 3% water agar^8^. Asci were dissected with a micromanipulator, and individual ascospores were isolated from the apex of the ascus. Ascospores were transferred to the opposite end of the agar-coated slide and placed at a distance apart from each other in the order of their original arrangement in the ascus and allowed to germinate. After 12 h, the germinated spores were transferred to PDA plates under a dissecting microscope and maintained as individual strains.

### Genome sequencing and *MAT* locus re-sequencing

The genome of the L mating type strain 06CWM-G22 was sequenced using the PacBio RS II on eight SMRT cells at University of Washington and assembled at the Washington State University. After identifying the contig where the *MAT* locus was located (see *In silico* analyses below), 13 primers pairs were designed based on the draft genome sequence to amplify and cover the entire locus including the flanking genes *APN2* and *SLA2* (see Supplementary Fig. S1 and Supplementary Table S2). These 13 primer pairs were used in PCR amplification of strain G22 of L mating type and strain G27 of S mating type. The following PCR cycling conditions were used for each of the 13 primer pairs: initial denaturation (94 °C for 4 min); 35 cycles of denaturation (94 °C for 30 s), annealing (55 °C for 30 s), and extension (68 °C, 30 s); final extension (68° for 8 min); hold (15 °C). Reaction mixtures were 20 μL in volume and contained 1.0 × of 10 × PCR buffer, 1.5 mM MgSO_4_, 0.5 mM total dNTPs, 0.5 μM primer (each), 0.5 U Taq, and 2 μL of 10 ng/μL template DNA. The PCR products were checked using 1% agarose gel electrophoresis (IBC BioExpress), purified with ExoSAP-IT and sequenced using corresponding primers at the Washington State University Bioinformatics Core, Pullman, WA, USA. The overlapping sequences from the PCR amplicons were assembled and analyzed in CLC Genomics Workbench.

### *In silico* analyses

To identify the contig in the draft genome that contained the *MAT* locus, the *MAT* locus (JQ815884) of *S. sclerotiorum* was used as query to search the draft genome sequence of isolate G22 using the CLC Genomics Workbench. The *MAT* coding and intron positions of *S. trifoliorum* were predicted using the *de novo* AUGUSTUS online interface. For AUGUSTUS, predictions were derived from the available model that was trained on *Botrytis cinerea*. Protein domains were identified with searches of the full pfam database V27.0 (http://pfam.xfam.org/). To detect the repeat sequence in the *MAT* locus sequences of G22 and G27, Tandem Repeat Finder was used to examine the *MAT* locus sequences against themselves^29^.

### Comparison of the *MAT* locus and gene arrangements

To determine the L and S alleles of the *MAT* locus in multiple isolates, a pair of primers (4F/6R) was selected from the 13 primer pairs to flank the sequence of the deletion region. All isolates used in this study were subjected to PCR amplification with the primers. The PCR conditions are same as described above. The PCR product sizes in L and S alleles were about 3.8 kb and 0.8 kb, respectively. The PCR fragments were checked by agarose gel electrophoresis and PCR products of representative isolates were sequenced as described above to confirm sequence identity.

### Gene Expression analysis

RT-PCR assays were used to monitor the expression of *MAT* genes in strain G22 of L mating type and strain G27 of S mating type. First-strand cDNA synthesis was prepared from DNase-treated total RNA using the iScript cDNA Synthesis Kit (Bio-Rad) following the manufacturer’s instructions. The house keeping gene *β-tubulin* was included as a positive control. Five primer pairs for four *MAT* genes (MAT1-1-5, 5′ truncated MAT1-1-1, MAT1-2-1, MAT1-2-4 and 3′ truncated MAT1-1-1) were listed in Supplementary Table S3. The RT-PCR primer pairs for transcripts of MAT1-1-5, 5′ fragment of MAT1-1-1 and MAT1-2-1 each span an intron, to allow detection of potential DNA contamination and to confirm correct intron splicing by sequencing. The RT-PCR products for each gene was checked by electrophoresis and also by sequencing using the corresponding PCR primers to confirm the gene-specific amplification and appropriate intron locations.

## Additional Information

**How to cite this article**: Xu, L. *et al.* Direct repeat-mediated DNA deletion of the mating type *MAT1-2* genes results in unidirectional mating type switching in *Sclerotinia trifoliorum*. *Sci. Rep.* **6**, 27083; doi: 10.1038/srep27083 (2016).

## Supplementary Material

Supplementary Information

## Figures and Tables

**Table 1:** Comparison of *MAT* genes of *Sclerotinia trifoliorum* with corresponding genes in *S. minor*^16^, *S. sclerotiorum*^17,18^ and *Botrytis cinerea*^18^.

Gene	Species	Strain	GeneBank/Broad Institute accession number	Gene length (bp)	Nucleotide identity (%)	No. of introns	Coding Sequence length (bp)	Protein size(aa)	Amino acid identity (%)
*MAT1-1-1*	*S. trifoliorum*	G22	–	–	–	–	–	–	–
	*S. trifoliorum*	G27	KU726097	1212	–	2	1110	370	–
	*S. minor*	SM1	KC894718	1106	89.7	2	1005	335	81.1
	*S. sclerotiorum*	44Ba1	JQ815883	1106	88.7	2	1005	335	78.2
	*S. sclerotiorum*	1980	XM_001594147.1/SS1G04004	826	88.5	1	777	259	77.1
	*B. cinerea*	B05.10	XM_001546388.1/BC1G_15148	1161	77.4	2	1062	354	69.9
*MAT1-1-5*	*S. trifoliorum*	G22	KU726096	1306	–	3	1131	377	–
	*S. trifoliorum*	G27	KU726097	1306	100	3	1131	377	100
	*S. minor*	SM1	KC894718	1303	95.4	3	1131	377	92.3
	*S. sclerotiorum*	44Ba1	JQ815883	1303	94.7	3	1131	377	91.2
	*S. sclerotiorum*	1980	XM_001594146.1/SS1G04003	1303	94.8	3	1131	377	91.2
	*B. cinerea*	B05.10	XM_001546387.1/BC1G_15147.1	1301	77.3	3	1131	377	76.3
*MAT1-2-1*	*S. trifoliorum*	G22	KU726096	1210	–	2	1098	366	–
	*S. trifoliorum*	G27	–	–	–	–	–	–	–
	*S. minor*	SM1	KC894718	1289	94	2	1185	395	89.6
	*S. sclerotiorum*	44Ba1	JQ815883	1289	93.5	2	1185	395	89.6
	*S. sclerotiorum*	1980	XM_001594149.1	1289	93.5	2	1185	395	89.9
	*B. cinerea*	T4	FQ790352.1	1248	78.3	2	1143	381	76.5
*MAT1-2-4*	*S. trifoliorum*	G22	KU726096	925	–	1	879	293	–
	*S. trifoliorum*	G27	–	–	–	–	–	–	–
	*S. minor*	SM1	KC894718	941	92.6	2	843	281	73.2
	*S. sclerotiorum*	44Ba1	JQ815883	944	92	2	846	282	73.6
	*S. sclerotiorum*	1980	XM_001594148.1/SS1G04005	944	92	2	846	282	63.9
	*B. cinerea*	T4	FQ790352.1	1517	42.6	3	1230	409	50.7

**Table 2:** Comparison of *MAT* intergenic spacer regions in *Sclerotinia trifoliorum* self-fertile isolate 06CWM-G22 with those of Inversion positive strains of *S. minor* (SM24) and of *S. sclerotiorum* (44Ba12).

Strain	APN2 –.*MAT1-1*-5	*MAT1-1*-5–5′ fragment of *MAT1-1-1*	5′ fragment of *MAT1-1-1*–*MAT1-2-1*	*MAT1-2-1*–*MAT1-2-4*	*MAT1-2-4*–3′ fragment of *MAT1-1-1*	3′ fragment of *MAT1-1-1*–SLA2
G22	2917	2851	12	499	177	1292
SM24	1699	406	0	467	507	616
44Ba12	2897	406	0	489	507	624
