# Supplementary material for: Direct repeat-mediated DNA deletion of the mating type MAT1-2 genes results in unidirectional mating type switching in Sclerotinia trifoliorum
Source: Sci Rep. 2016 Jun 3;6:27083. doi: 10.1038/srep27083 (PMC4891775; doi:10.1038/srep27083)
Supplement: Supplementary Information [file srep27083-s1.pdf]

Direct repeat-mediated DNA deletion of the mating type *MAT1-2* genes results in unidirectional mating type switching in *Sclerotinia trifoliorum*

Liangsheng Xu[1], Teresa M. Jardini[1] and Weidong Chen[1,2*]

[1] Department of Plant Pathology, Washington State University, Pullman, WA 99164, USA.

[2] USDA-ARS, Grain Legume Genetics and Physiology Research Unit, Washington State University, Pullman, WA 99164, USA.

Supplementary Table S1. Isolates of *Sclerotinia trifoliorum* used in the study with their geographic location, PCR production amplified by the specific primer 4F and 6R and *MAT* genotype

Isolate	Year	Location	*MAT* allele[a]	Heterokaryons[b]
05WM-6	2005	Robbins, CA	L	No
05WM-8	2005	Colusa, CA	L	Yes
05WM21	2005	Five Points, CA	S	No
06CWM-A8	2006	Farmington, CA	L	No
06CWM-C4	2006	Stockton, CA	L	Yes
06CWM-D5	2006	Davis, CA	S	No
06CWM-F2	2006	Farmington, CA	S	No
06CWM-F6	2006	Knights Landing, CA	S	No
06CWM-F9	2006	Knights Landing, CA	S	No
06CWM-G2	2006	Five Points, CA	L	No
06CWM-G7	2006	Five Points, CA	L	Yes
06CWM-G22	2006	Five Points, CA	L	No
06CWM-G23	2006	Five Points, CA	S	No
06CWM-G27	2006	Five Points, CA	S	No
06CWM-G34	2006	Five Points, CA	L	No
06CWM-G39	2006	Five Points, CA	L	Yes
06CWM-G48	2006	Five Points, CA	L	No
06CWM-G55	2006	Five Points, CA	L	Yes
06CWM-H2	2005	Five Points, CA	L	No
06CWM-H6	2006	Five Points, CA	L	No
TP05-06	2006	Prosser, WA	S	No
WMAL-1a	2005	Visalia, CA	S	No

[a] *MAT* L allele refers to the allele found in the self-fertile isolates derived from large ascospores, and S allele the allele found in the self-sterile isolates derived from small ascospores

[b] Heterokaryon refers presence of both alleles always a strong L allele and a faint S allele.

Supplementary Table S2. PCR primers used to amplify the entire MAT locus including the flanking genes APN2 and SLA2 (See Supplementary Figure S1 for primer locations on the *MAT* locus) along with their sequences used in this study

Primer	Sequence (5´-3´)	Usage[a]
1F	CAAGTATGTCACAAAGCTCACA	*MAT* locus
1R	GATGGGGTTGCATTCTGATTAC	
2F	TTTGGATGGAGAAATTGATGCC	*MAT* locus
2R	AGTGATGTAAGAAGCTCAAGGT	
3F	AGGAAGAGATTAGGTGAGATGC	*MAT* locus
3R	TGTAACCCAATTCGATCGATTC	
4F	ATGCCTCGTTGAAGTCTAAAAC	*MAT* locus
4R	CAATTAGCTGCAGGTCAAAGAA	
5F	CTCTTATAGCCAAAGTTTCGCA	*MAT* locus
5R	GATAAGACCGTACTACAGAGCA	
6F	AAGGAGATTTATGATGCGATGC	*Es MAT* locus
6R	ATCTTCCGCAAAAGTACAAGTC	
7F	ATTTGGTATGAAAAGGATCGCC	*MAT* locus
7R	CACGTAATGATGAGCTCTAGGA	
8F	TATCCTCTAAGATGATCGTCCC	*MAT* locus8
8R	TCGATTAACCTTCTTCAAGATT	
9F	TCTTGAAGAAGGTTAATCGATAA	*MAT* locus
9R	CTGCGAGATGTTATAGTCTTGA	
10F	AGGCATCACAGGTAAAGAATTT	*MAT* locus
10R	GTAATCAACCTCGAAATGAAGC	
11F	CTTCGGTGATACTGTGTTATGT	*MAT* locus
11R	AAAATTATCAAGGGCCTGCG	
12F	CGCAGGCCCTTGATAATTTT	*MAT* locus
12R	ATTTTCAAACATGCACACACAT	
13F	ATGTGTGTGCATGTTTGAAAAT	*MAT* locus
13R	AGAGGAGCAACTAAAGAAACAG	

.

Supplementary Table S3. PCR primers used in RT-PCR

Primer	Sequence 5′-3′	Target gene	DNA size (bps)	RNA size (bps)
M115F	CCATTACCCACCGAGTATG	*MAT1-1-5*	658	712
M115R	TCCTCAAGATCCCATTTTGG			
M111F	CTCAGTACCGCGTGTCAAAA	*5' MAT1-1-1*	231	280
M111R	GCGGAAGATTTCCAAGCATA			
M121F	TTCCTGGATTGTCGAAGGAG	*MAT1-2-1*	450	490
M121R	GATCTTTGGTTTGGGAGCAA			
M124F	GACGGGACGGTCTCAGATTA	*MAT1-2-4*	297	297
M124R	TGAGAAGCCCTGTTTCGAGT			
M111F1	ATGAAGCGGCAGCAAAGAAAG	*3' MAT1-1-1*	370	370
M111R1	AGAAAGTGATGATGTCCAAATC			

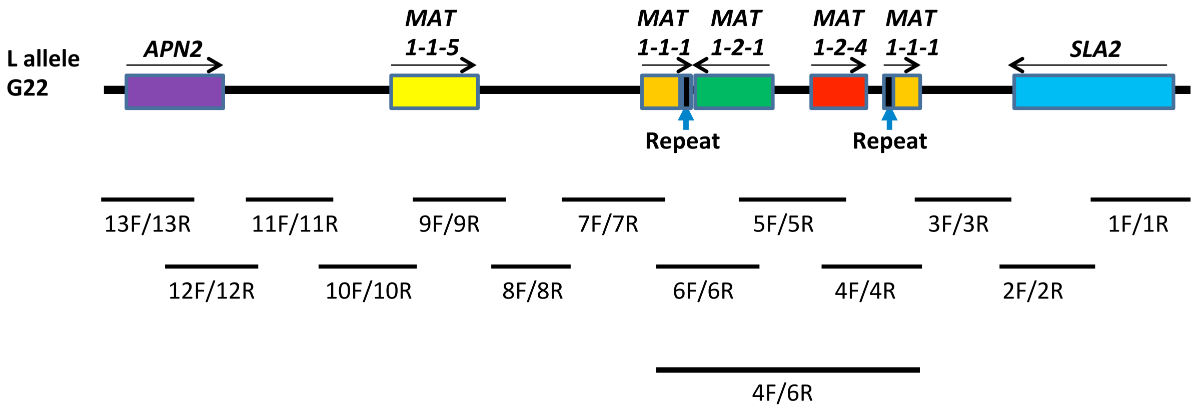

Supplementary Figure S1. Locations of the overlapping PCR primers used to amplify the entire *MAT* locus of *S. trifioliorum*.

MAT1-1-1 Alpha box domain

STG22	NSYILFRTFYMILTMLGNLPQKYKSSILSILWRGDPFHTKWSILARAYTLMR
SM1	NSYILFRTFYMILNVLGNIPQKYKSAILSVLWGRDPFHAKWSILARAYTLMR
SS44Ba1	NSYILFRTFYMILNVLGNIPQKYKSAILSVLWGRDPFHAKWSILARAYTLMR
SS1980	----------MILNVLGNIPQKYKSAILSVLWGRDPFHAKWSILARAYTLMR
BC	NSYILFRTFYMILTVLGNIPQKYKSAILSVLWGRDPFHAKWSIIARAYTLMR

MAT1-2-1 HMG-DNA binding domain

STG22	IPRPANEWILYRADNHLPIKKAYPGITNNEISSIIAGMWAAETPERRLKYKI
SM1	IPRPANEWILYRADNHLPIKKAYPGITNNEISSIIAGMWAAETPERRLKYKI
SS44Ba1	IPRPANEWILYRADNHIPIKKAYPGITNNEISSIIAGMWAAETPERRLKYKI
SS1980	IPRPANEWILYRADNHIPIKKAYPGITNNEISSIIAGMWAAETPERRLKYKI
BC	IPRPANEWILYRADNHLPIKKAYPGITNNEISSIIAGMWAAETPERRLKYKI

Supplementary Figure S2. Alignment of amino acid sequences of the alpha box of *MAT1-1-1* and high mobility group (HMG)-DNA binding domain of *MAT1-2-1* of *Sclerotinia trifoliorum* (STG22), *S. minor* (SM1) *S. sclerotiorum* (SS44Ba1 and SS1980) and *Botrytis cinerea* (BC).
